# The UK prosthetic and orthotic workforce: current status and implications for the future

**DOI:** 10.1186/s12960-023-00882-w

**Published:** 2024-01-08

**Authors:** Nicola Eddison, Aoife Healy, Enza Leone, Caroline Jackson, Bracken Pluckrose, Nachiappan Chockalingam

**Affiliations:** 1 Centre for Biomechanics and Rehabilitation Technologies, Science Centre, Staffordshire University, Leek Road, Stoke on Trent, ST4 2DF United Kingdom; 2 Royal Wolverhampton NHS Trust, Wolverhampton, WV10 0QP United Kingdom; 3 DM Orthotics Ltd, Unit 2, Cardrew Way, Cardew Industrial Estate, Redruth, Cornwall TR15 1SS United Kingdom; 4 Blatchford Clinic, Unit D, Antura, Kingsland Business Park, Basingstoke, RG24 9PZ United Kingdom

**Keywords:** Orthotist, Prosthetist, Orthotics, Prosthetics, Orthotic devices, Prostheses and Implants, NHS, Workforce planning, Health Workforce

## Abstract

**Background:**

Prosthetists and orthotists (POs) are the smallest of the 14 allied health profession (AHP) workforces within NHS England. Obtaining data on the workforce has always been challenging due to this information being held across different organisations. An understanding of the prosthetic and orthotic (P&O) workforce is essential to ensure that it is adequately equipped to meet the evolving needs of users of P&O services. The study aims to estimate the size and composition, for the first time, of the UK P&O workforce and P&O service provision.

**Methods:**

To gather the required information, two surveys (one for the UK P&O workforce and one for UK P&O private company) and two freedom of information (FOI) requests [one for all NHS Trusts and Health Boards (HB) in the UK and one for the higher education institutes in the UK offering programmes leading to registration as a PO were developed and distributed from September to December 2022.

**Results:**

The P&O workforce survey received a 74% response rate (863 POs) and 25 private companies reported employing one or more P&O staffing groups. From the FOI requests, 181 of a potential 194 Trusts/Health Boards and all four higher education institutions responded. The study indicated a total of 1766 people in the UK P&O workforce, with orthotists and orthotic technicians representing the largest percentage of the workforce at 32% and 30%, respectively. A greater percentage of prosthetists (65%) and orthotists (57%) were employed by private companies compared to the NHS. Only 34% of POs stated that they “definitely” planned to remain in the workforce for the next 5 years. The current UK PO employment levels are 142 to 477 short of the World Health Organisation’s (WHO) recommendation.

**Conclusions:**

The low job satisfaction amongst many POs and the projected increase in the number of people who will require prosthetic and/or orthotic care in the UK are challenges for future UK P&O services. Strategies are required to create a sustainable and resilient workforce that can meet the needs of a changing healthcare landscape.

## Introduction

The prosthetic and orthotic (P&O) workforce consists of individuals who are specialised in assessing, designing, manufacturing, and fitting orthotic/prosthetic devices. The UK P&O workforce has three categories: prosthetist/orthotist (PO), P&O technicians, and P&O support workers. In the UK, PO is a protected title requiring registration with the Health and Care Professions Council (HCPC) to practice; as of 4 October, 2023, there are 1174 registered POs. POs are the smallest of the 14 allied health profession (AHP) workforces within National Health Service (NHS) England; for comparison, there are 71 316 physiotherapists, 45 222 occupational therapists, 18 420 speech and language therapists, and 12 250 podiatrists registered in the UK [1]. There has been a 68% increase in the number of registered POs from 699 in 1999 to 1174 in 2023, with an average annual change of + 2% (ranging from − 3% to + 7%) [1–4]. P&O technicians design and manufacture custom-made devices to meet the specifications/prescriptions determined by the PO. Whilst specific qualifications are not required to work as a P&O technician in the UK, in 2022 an apprenticeship programme to qualify as a Level Three Prosthetic and Orthotic Technician was launched. P&O support workers work with POs to deliver patient care, working under a range of supervisory arrangements which sometimes include guidelines and frameworks. Unlike POs, P&O technicians and support workers are not required to register with a regulator, so no information on the numbers within these staffing groups exists. Both clinical, technical, and support staff work closely with service users to understand their unique needs.

As outlined in the recently published NHS Long-Term Workforce Plan [5], increased numbers of healthcare support workers (a term that covers a variety of health and care support roles, including P&O support workers) will be needed to meet the expected growth in demand for healthcare services. The role of P&O support workers within the P&O workforce is relatively recent and prior to the launch of the Allied Health Professions’ Support Worker Competency, Education, and Career Development Framework [6] in 2021, they were trained to localised frameworks with no national standardisation.

To train as a PO in the UK, two universities offer a BSc honours degree in prosthetics and orthotics. In 2021, a level 6 apprenticeship course leading to a BSc (Honours) prosthetics & orthotics degree/degree apprenticeship was started, and in 2022 an MSc in prosthetics & orthotics commenced. The PO programmes cover both orthotics and prosthetics, thus graduates are dual qualified, but most specialise in prosthetics or orthotics. The UK P&O workforce is employed by both the NHS and the private sector. Whilst the NHS plays a significant role in delivering these services by employing the clinicians directly (termed in-house services), it also procures contracts with private companies to provide NHS P&O services, and private companies also treat private patients, resulting in a complex employment landscape.

The data available from the HCPC only provide information on the number of registered POs and do not capture how many of these POs are currently in the workforce. Obtaining comprehensive data on the workforce have historically been challenging due to this information being held across different organisations. Reports to date from various organisations [7–12] have provided limited information on the workforce and highlighted the challenges in sourcing information on the workforce. NHS England’s 2015 report [9] on improving the quality of orthotic services in England identified four workforce challenges, which to date have not been fully addressed: (1) the lack of data, as orthotists employed by the private sector did not have a unique occupation code for use in NHS electronic staff record systems, (2) the demand for orthotists was likely to increase due to the ageing population and rising prevalence of non-communicable diseases, (3) the requirement to address the lack of accurate workforce planning data to enable planning for the future, and (4) the need to encourage and consider a multidisciplinary team approach in orthotic services to reduce waiting times and improve orthotics services.

Staff retention issues are a growing concern for P&O services. The 2023 HCPC report [13] on attrition rates concluded that 12.8% (1 in 8) of POs had left the HCPC register within 4 years of their first registration. This rate was the highest amongst all the allied health professions on the HCPC register; for comparison, paramedics reported the lowest rate with 1 in 56 having left the register, and across the professions around 1 in 18 left during this time. This issue is further confounded by staffing issues, a recent article [14] stated that the orthotic profession within England and Wales may be facing a staff retention crisis, with 37% of the orthotists indicating that they would leave the profession if they could.

Understanding the P&O workforce is essential to ensure that it is adequately equipped to meet the evolving needs of users of P&O services. This will facilitate informed decision-making, resource allocation, and strategic planning to ensure that service users receive timely and effective P&O interventions. Therefore, this paper aims to (1) estimate the size and composition of, for the first time, UK P&O service provision and the P&O workforce, (2) explore the role of P&O support workers within the workforce, (3) explore job satisfaction of POs, and (4) estimate the number of newly qualified POs joining the workforce.

## Methods

The information presented in this paper is part of a wider project which aimed to profile the UK prosthetic and orthotic workforce and project the workforce for the twenty-first century [15].

As the information on the P&O workforce is held across different organisations, we were required to utilise a range of data capture methods to ensure completeness of information. To gather the required information to create a detailed picture of the UK prosthetic & orthotic (P&O) workforce two surveys were devised; (1) a survey for the UK P&O workforce, and (2) a survey for the UK P&O private companies who employ P&O staff. Both surveys were produced using the online survey platform Qualtrics (Qualtrics International, USA; www.qualtrics.com). The P&O workforce survey was distributed via social media, prosthetic and orthotics networks, The British Association of Prosthetists and Orthotists (BAPO), the British Healthcare Trades Association (BHTA), the 2022 UK International Society for Prosthetics and Orthotics (ISPO) conference, NHS Orthotic Managers Group (NOMAG), Health Education England contacts, University of Salford, Keele University, the University of Strathclyde, the University of Derby, and student representatives. A suite of videos from influential members of the P&O profession and stakeholders was created to help promote the survey via social media. The survey was open to all UK-based prosthetists/orthotists (POs), P&O technicians, P&O support workers, and PO students/apprentices. The P&O private companies’ survey was sent directly to known stakeholders and distributed via a dedicated social media campaign. In addition, freedom of information (FOI) requests were sent to every NHS Trust and Health Board (HB) in the UK and each higher education institute in the UK offering programmes leading to registration as a PO (University of Salford, University of Strathclyde, Keele University, and the University of Derby). Freedom of Information Act [16] requests were utilised as they provide access to information held by public authorities. The information collected within the surveys and FOI requests is presented in Table 1. Responses to the surveys and FOIs were collected from September to December 2022, with analysis conducted using SPSS Statistics, version 28.

**Table 1:** Requested information from surveys and freedom of information requests

Survey		Freedom of Information request	
P&O workforce	Private P&O companies	UK NHS Trusts/Health Boards	Higher Education Institutes providing P&O programmes
• Demographics • Plans for further education • Career goals • Skills–current and requirements for the future • Populations they treat • Employment, employer, working hours, work settings, salary • Job satisfaction	• Which P&O staff groups they employ • Number of employees • Staffing data for the previous 5 years • Number of current vacancies • Vacancies data for the previous 5 years • Recruitment issues	• Which P&O staff groups they employ • Number of employees • Staffing data for the previous 5 years • Number of current vacancies • Vacancies data for the previous 5 years • Recruitment issues	• Number of staff and their level of education within each of the P&O staffing groups • Number of current students/apprentices on PO programme leading to HCPC registration • Student/apprentice demographics • Number of students/apprentices on the programme (previous 5 years data) • Skills and knowledge included in the curriculum • Number of applicants over the last 5 years • Capacity of the programme and plans for expanding number of students accepted on to the programme (previous 5 years data) • Number of students who have graduated from the programme (previous 5 years data)

## Results

A total of 905 responses to the P&O workforce survey were received, with most responses (641; 71% of responses) received from POs; 419 orthotists, 177 prosthetists, and 45 dual practising POs. The remaining responses were from P&O technicians (99), support workers (16), P&O apprentices (9), and students (140). At the time of the survey, there were 1164 HCPC registered POs [17], and 633 of the 641 POs were HCPC registered, equating to 54% of HCPC registered POs completing the survey. Findings, from the P&O private companies’ survey and the FOI requests to NHS Trusts/HBs, indicated that 863 POs were working as clinicians in the UK, therefore our survey response rate was 74% of HCPC registered POs.

Forty-eight private P&O companies (including all the known large employers) responded to the survey, with 25 companies reporting employing one or more P&O staffing groups. A total of 181 responses, out of a potential 194, were received from the FOI requests to UK NHS Trusts/HBs, equating to a response rate of 93.29%, and responses were received from all four higher education institutions.

### Size and composition of UK P&O service provision and the P&O workforce

#### P&O service provision

**Fig. 1:**
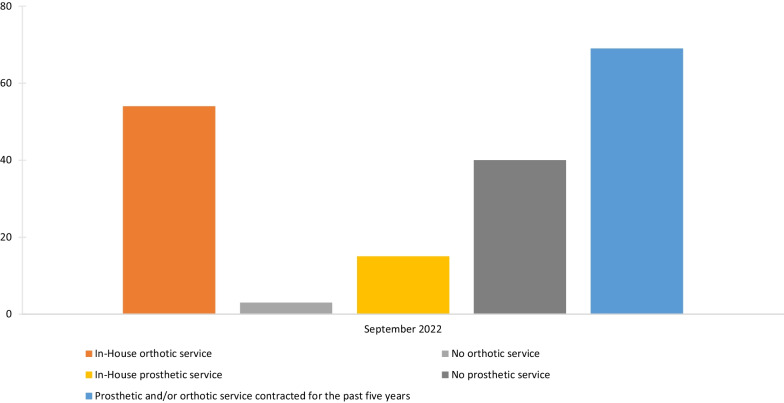
Number of NHS in-house orthotic and prosthetic services

Based on the responses from the freedom of information requests to the NHS Trusts/HBs (Fig. 1), the NHS had a total of 54 in-house orthotic services; 41 (76%) in England, eight (15%) in Scotland, five (9%) in Wales and none in Northern Ireland. With 15 in-house prosthetic services; seven (47%) in England, five (33%) in Scotland, three (20%) in Wales, and none in Northern Ireland. Sixty-five of the 181 Trusts/HBs who responded (36%) reported that they have directly employed one or more of the prosthetic and orthotic staffing groups within the last 5 years. Forty of the 181 Trusts/HBs (22%) reported that they do not currently have or have had a prosthetic or orthotic service in the last 5 years. Seventy-six of the 181 Trusts/HBs (36%) reported their prosthetic and/or orthotic service had been contracted to an external supplier for the past 5 years. The number of NHS services that currently have an in-house service reported that this model has remained relatively static over the last 5 years.

#### P&O workforce and vacancies

**Fig. 2:**
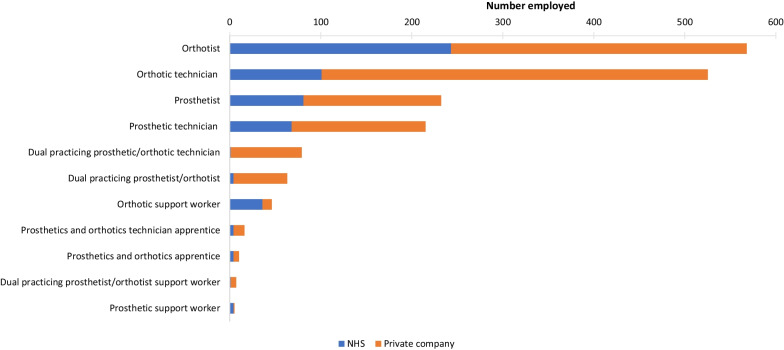
Number of people employed in the UK P&O workforce

From the survey responses of private P&O companies and the freedom of information requests to NHS Trusts/HBs, the number of people employed in the P&O workforce (see Fig. 2) and vacancies were determined. There was a total of 1766 people in the P&O workforce, with orthotists and orthotic technicians representing the largest percentage of the workforce at 32% and 30%, respectively. A greater percentage of prosthetists (65%) and orthotists (57%) were employed by private companies compared to the NHS. The data showed that 35% of all P&O staff in the private sector were employed by four private companies.

**Fig. 3:**
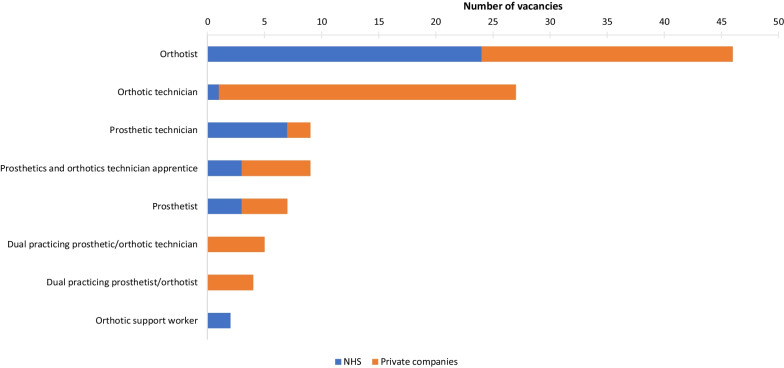
Number of vacancies in the UK P&O workforce

There were 109 vacancies in the P&O workforce (Fig. 3). The NHS Trusts/HBs reported a total of 40 vacancies across 23/68 in-house services, with vacancies reported across various regions of England, Scotland, and Wales. The private companies reported a total of 69 vacancies across 14/25 companies. The highest number of vacancies was for orthotists (46), followed by orthotic technicians (27). A high percentage of both NHS Trusts/HBs and private companies who had vacancies reported that they had been unable to recruit, 43% and 57%, respectively. One Trust/HB and three private companies reported using international recruitment agencies for the P&O workforce. Five of the NHS P&O workforce and two of the private sector P&O workforce had been recruited from overseas.

### Role of P&O support workers within the workforce

Within the P&O workforce survey, five P&O support workers and 108 POs responded that they thought the role of P&O support workers could be better utilised. They were then asked to list the skill(s) that they thought were not currently utilised, with three support workers and 74 POs responding to this question. Support workers felt they had clinical skills and skills related to outcome measures and research that were not being utilised. The POs' responses agreed with this, two-thirds of all responses reported that support workers' clinical and research skills are not being utilised within their service. An example response stated that support workers can be better utilised in “fitting basic devices such as post-op knee braces, gaiters, stock footwear, and insoles”. Comments also discussed extending the scope of support workers to include clinically related jobs including collection of outcome measures, telephone reviews, patient handling/casting assistance, educating patients on donning/doffing orthoses, and guidance on using orthoses. One responder referenced that ‘assistants would find the job more fulfilling if they were in clinic more and developed clinical skills’ which links to job satisfaction and the scope of the support workers’ job. Other skills reported which are not currently utilised include providing public or mental health support, triaging, and technical skills including adjustments or repairs.

### Job satisfaction of POs

**Fig. 4:**
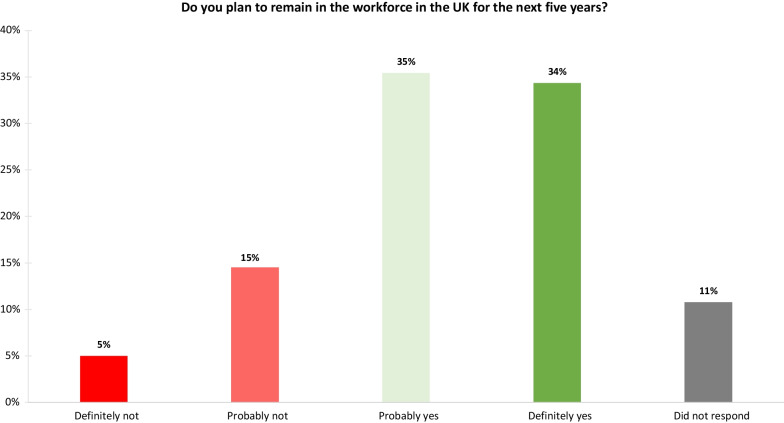
POs responses to remaining in the profession for the next 5 years

**Fig. 5:**
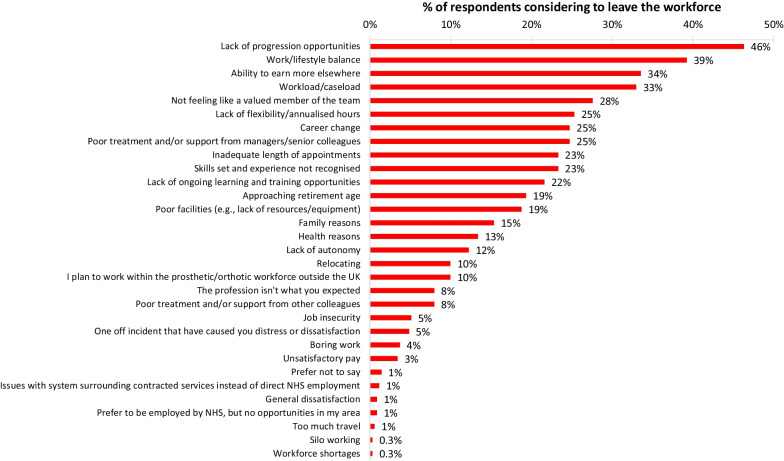
Reasons POs were considering leaving the workforce

Within the P&O workforce survey job satisfaction was explored, participants were asked if they planned to remain in the workforce for the next 5 years; with only 34% stating that they “definitely” planned to remain in the profession (Fig. 4). Those who selected “probably yes”, “probably no”, and “definitely no”, a total of 352 respondents, were asked to provide the reason(s) why they were considering leaving the P&O workforce (Fig. 5). A total of 31 different reasons for considering leaving the profession were provided; the five reasons with the highest responses were lack of progression opportunities, work/lifestyle balance, ability to earn more elsewhere, workload/caseload, and not feeling like a valued member of the team.

### Number of newly qualified POs joining the workforce

With information gained from freedom of information requests to the universities, the average number of PO graduates annually over the previous 5 years was 53 (range 47–58); this information is based on two of the four programmes, as two programmes which started in 2021 and 2022 have not had any graduates yet. The reported maximum number of students that the institutions could accept into their PO programmes for 2022/2023 was 93 (this does not include the capacity of the Prosthetic and Orthotic degree apprenticeship which was not provided).

From the P&O workforce survey, responses were gathered from 140 university students studying to qualify as a PO; this accounts for 53% of the known PO students, and six apprentices studying to qualify as a PO, 60% of the known PO apprentices. Most students and apprentices (92%) reported that they planned to register as a PO in the UK after graduation. Of those respondents who were not planning to register as a PO, eight reported that they were international students who wished to return to their home countries to work or wished to work internationally; one was interested in a research career; and one was unhappy with UK pay and conditions. In terms of career goals, the most popular responses were to work as a prosthetist (41%), a dual practising PO (39%), and an advanced clinical practitioner or consultant PO (39%). In addition, 66% of students and apprentices reported that they would like to work in the NHS. Whilst the workforce needs for well-established professions such as medicine, nursing, dentistry, and pharmacy have been thoroughly examined, and their graduate-to-stock ratios are available [18, 19], comparing the number of training positions for prosthetics and orthotics in the UK is challenging. These larger professions have substantial figures, and their overall requirements are discussed at governmental and higher strategic levels. In contrast, smaller professions like prosthetics and orthotics address their needs at operational levels. It is also important to note that the total training places for Allied Health Professions programs in the UK are overseen by the NHS.

## Discussion

This study was the first to explore the entire UK P&O workforce across all sectors. The UK PO workforce is of limited size and as it is further sub-grouped into three distinct categories, 66% work as orthotists, 27% as prosthetists, and 7% as dual practising PO, it shows the limited size of each professional group.

International comparative data are needed to establish accurate figures on the number of POs globally and PO numbers should be determined by country-specific factors (e.g. the need, the healthcare system, skills of personnel, and type and range of products), The World Health Organisation (WHO) has reported that in some high-income countries there are 15–20 POs per million population [20] which would equate to 1005–1340 POs for the UK population. The current number employed in the UK (as identified from responses to the private companies’ survey and the FOI requests to NHS Trusts/HBs) is 863 and therefore falls 142 to 477 POs below this range. Due to the limited size of the PO workforce, a small shortage in POs can have a large impact on P&O service provision. Our findings are consistent with previous reports on the UK PO workforce [7–10], and the UK is not alone in this issue of having a lower number of POs than is needed for its population. Reports from Australia in 2021 [21] and the USA in 2015 [22] have highlighted the requirement for more POs in their countries.

Although private companies are fewer in number than NHS trusts/HBs, they employ the majority of the P&O workforce. A little over 20% of NHS trusts/HBs reported not having orthotic or prosthetic services, which may make it hard for clinicians in some regions to find local employment and potential challenges for service users in those areas to access P&O services. A small number of employees and a workforce that has been divided by employer type (NHS and private sector) into many small competing groups are likely to lead to silo working patterns, and reduced fluidity of services for service users moving from one area to another. Service delivery partnerships are evidenced to create these silos as well as prioritisation of different outcomes and reduced crosslinking between services [23]. Three of the five main reasons POs were considering leaving the profession are related to this point: lack of progression opportunities, workload/caseload, and not feeling like a valued member of the team. Progression opportunities are likely to be significantly less if you work in a small team, or a widespread company [23]. It may be hard to balance your workload without support workers and PO support available daily, and it is likely much harder to feel like a valued team member when you work alone. Improved unity across the UK's services may have a direct impact on attrition for these reasons. A quarter of POs selected a lack of flexibility/annualised hours as a reason for considering leaving the profession, this could potentially be attributed to a lack of flexibility in employment contracts with most POs having full-time contracts and working clinically 80–100% of their time. Our findings on job satisfaction are consistent with previous research [14] and highlight a significant challenge for the PO workforce.

Similar professions in other countries have witnessed challenges leading to attrition. In Australia, the National Disability Services Workforce Census Report found difficulties in recruiting and retaining allied health workers and increased staff turnover [24]. Pay and conditions, limited training and development opportunities, and lack of support and supervision in workplaces were identified as issues affecting people entering and remaining in the sector [25]. Exploring these factors within the UK workforce could provide insights into retention challenges and inform strategies to mitigate attrition.

Support workers have been shown to positively impact allied health professional services in England [26] but are underused in P&O services. NHS England has pledged to build a bigger group of support workers in their long-term workforce plan [5]. Support workers reported that they have clinical skills that are currently unutilised. Wider availability of support workers, who are given greater responsibility, could help reduce the workload for POs. Considering that a high workload is one of the reasons why P&O professionals are considering leaving the profession, this may have the potential to reduce the attrition rate.

The recently published NHS long-term workforce plan [5] supports the training of POs through apprenticeships with a 10-year goal to increase the proportion of POs joining the AHP workforce from an apprenticeship route to 25–50%. Barriers to traditional recruitment include the location of universities providing BSc PO training, with none in the southern part of the UK, one in the north of England and the other in Scotland, as well as no guarantee of jobs in specific UK regions following graduation. Apprenticeships aim to overcome these by allowing the apprentice to live and work in their local area. POs may still face location barriers due to the limited number of jobs in each region. Whilst there are high current vacancies (109 across the UK), suggesting sufficient opportunity to grow the workforce in the coming years, these positions may be challenging to fill due to their location, employment type (NHS or private company), whether the type of position is suitable for the PO (whether it is an orthotist or prosthetist position), and whether the PO has the required level of expertise for the position.

Whilst there was a high response rate for POs (74%) within the P&O workforce survey, limited data were collected from support workers (26% of the known workforce) and technicians (12%). Therefore, their data cannot be considered representative of these P&O staffing groups and further initiatives are needed that engage with these staffing groups to support workforce planning.

## Conclusions

This study is the first to provide comprehensive data on the UK P&O workforce and has identified areas which require further investigation, including:

1. initiatives to address the reasons POs are considering leaving the profession
2. understanding why PO vacancies are not being filled
3. increasing the availability of PO support workers to reduce the workload for POs
4. exploring an expansion of the PO support worker role.

The low job satisfaction amongst many POs identified in this study in combination with the projected increase in the number of people that will require prosthetic and/or orthotic care in the UK are challenges for future UK P&O services. Strategies to address the identified challenges within the P&O workforce are required to work towards creating a sustainable and resilient workforce which can meet the needs of a changing healthcare landscape.

## Data Availability

The datasets generated or analysed during this study are available from the corresponding author upon reasonable request.
